# Early‐onset neonatal sepsis due to *Streptococcus salivarius*: A case report

**DOI:** 10.1002/ccr3.6837

**Published:** 2023-01-11

**Authors:** Sakviseth Bin, Sethikar Im

**Affiliations:** ^1^ Neonatal Intensive Care Unit Calmette Hospital Phnom Penh Cambodia

**Keywords:** multi‐drug resistance, neonatal sepsis, newborn, *Streptococcus salivarius*

## Abstract

*Streptococcus salivarius*, the most predominant human oral microbiota, is rarely associated with neonatal sepsis. This report aims to describe a rare culture‐proven case of early‐onset sepsis in a 3‐day‐old boy with isolated high‐grade fever. *Streptococcus salivarius* was isolated in two consecutive hemocultures and unexpectedly resistant to the conventional empirical antibiotics.

## 1 INTRODUCTION

Early‐onset sepsis (EOS) remains a potential life‐threatening condition for newborns, with the incidence of culture‐proven sepsis of 1.08 cases per 1000 live births.^1^ The symptoms are non‐specific and appear during the first seven days of life.^2^ It is usually due to the intrapartum transmission of bacteria either from the aspiration of contaminated amniotic fluid or from the birth canal during vaginal delivery.^3^ The most frequent pathogens are Escherichia coli and Group B Streptococcus (GBS).^1^ However, neonatal sepsis can also be associated with other rare bacteria, including the viridans group streptococci (VGS). We are presenting a case of EOS in a febrile neonate due to multi‐drug‐resistant *Streptococcus salivarius*, isolated in two hemocultures. This α‐hemolytic is known as the most predominant microbiota colonizing the buccal mucosa and dorsal lingual epithelium.^4^ It is the least pathogenic VGS, and mostly considered as insignificant or contaminant.^5^ Identified in 1906, it was phenotypically similar to *Streptococcus bovis*, which is significantly linked to bacteremia and endocarditis.^6^ *Streptococcus bovis* was also reported to have caused sporadic cases of bacteremia and meningitis in children.^7^

## 2 CASE REPORT

An inborn, full‐term, male infant was born by vaginal delivery after an uneventful pregnancy, to an afebrile mother, 28‐year‐old, gravida 2 para 1. It was an imminent birth without perinatal asphyxia. There was neither history of suspected intra‐amniotic infection nor prolonged prelabor rupture of membranes. He was observed in Well‐Baby Nursery for 24 h due to meconium‐stained amniotic fluid and later discharged to maternal ward.

The boy was admitted to Special Care Nursery at day 3 of life (DOL 3). He was febrile (axillary temperature 40°C) and agitated but calmed by breastfeeding. Alert and active, he had good coloration and good muscle tone. There was neither seizure nor respiratory distress. Vital signs were stable: pre‐ductal SpO2 98% on room air. Physical examination was unremarkable, with an open and flat anterior fontanel. Per unit protocol, septic workup was done, and empirical antibiotherapy was initiated with cefotaxime (100 mg/kg/day) and ampicillin (150 mg/kg/day) for suspected EOS. The initial complete blood count (CBC) was normal: leukocytes 13.4 × 10^9^/L, absolute neutrophil count (ANC) 10 × 10^9^/L, and immature to total neutrophil ratio (I/T ratio) 0.05; hemoglobin and platelets were normal (Table 1). Nonetheless, C‐reactive protein (CRP) was elevated to 21.8 mg/L. Lumbar puncture (LP) was normal with leukocytes 02/mm^3^, albumin 0.85 g/L, and glucose 0.61 g/L; the culture was sterile. Chest x‐ray was normal.

**TABLE 1:** Laboratory investigations

	Day of life (DOL)					
	DOL 3	DOL 5	DOL 8	DOL 12	DOL 15	Follow‐up at DOL 30
Leukocytes (4–9 × 10^9^/L)	13.4	14.6	16.9	21.6	21	15.7
Hemoglobin (13–17 g/dL)	16.2	16	18.2	14.7	13.2	16.4
Platelets (150–450 × 10^9^/L)	266	126	79	295	337	218
C‐reactive protein (<5 mg/L)	21.8	31.8	28.9	26.8	12.4	5.9
Hemoculture	*S. salivarius*	*S. salivarius*			Negative	

Around 72 h later, *Streptococcus salivarius* was identified in the peripheral hemoculture drawn at admission. It was susceptible only to vancomycin (Table 2). The minimum inhibitory concentration (MIC) of ampicillin was 8mcg/ml. The repeated culture confirmed the same pathogen. With persistent fever, decreased platelets, and persistently high CRP, antibiotics were changed to vancomycin. The infant was afebrile about 48 h after the treatment. At DOL 15, platelets increased progressively, and CRP declined to 12.4 mg/L. Due to clinical improvement, normalized platelets and negativation of the third hemoculture, the antibiotic was used for 10 days, and the boy was discharged home at Day 20. A follow‐up, which was arranged 10 days after discharge (DOL 30) to control CBC and CRP, showed a good clinical and laboratory improvement.

**TABLE 2:** Antibiogram for *Streptococcus salivarius*

Specimen	Blood
Gram Stain	Gram‐positive Cocci
Culture and Identification	*Streptococcus salivarius*
Sensitivity	
Ampicillin	R
Ceftriaxone	R
Vancomycin	Sensible
Chloramphenicol	R
Erythromycin	R
Clindamycin	R
Quinupristin/dalfopristin	I
Ofloxacin	R

## 3 DISCUSSION

*Streptococcus salivarius* is uncommon in pediatric patients and even rarer in neonatal population. This is a rare case of culture‐proven EOS in a symptomatic neonate. In the literature, there are two case reports of possible neonatal sepsis linked to *S. salivarius*.

In 2014, Molinaro et al.^8^ described a case of *S. salivarius* bacteremia in a term neonate from a GBS positive mother, who had received two doses of clindamycin. There were no other maternal risk factors. The female infant was born vaginally. She was asymptomatic, with normal sepsis workup. However, *S. salivarius* was isolated in two consecutive blood cultures, so they decided to treat the infant with penicillin for 10 days.

Recently, Keerthi et al.^9^ reported an unusual presentation of neonatal pneumonia, presumptively due to *S. salivarius* at the chronological age of 44 days in an ex‐preterm infant of 35 weeks, presenting with fever and wheezing at the emergency department. However, we could not rule out the possibility of “contaminant” as it was identified in only a single hemoculture. Moreover, it was more likely to be a community‐acquired pneumonia, rather than a sepsis resulting from vertical transmission at birth.

In our case study, the infant was febrile, with elevated CRP. It was presumably not contamination since the infant was symptomatic, and *S. salivarius* was isolated in two consecutive cultures done at different sites each time. This sufficiently proves that Gram‐positive cocci are not always GBS, and VGS, though rare, should not be neglected because of its life‐threatening complications in some pediatric cases, including endocarditis and meningitis.^6^, ^7^ In response, LP was also performed at admission to rule out early‐onset meningitis, and bedside heart ultrasound showed normal heart structure with good left ventricular function and no vegetation, which ruled out endocarditis. In addition to these complications, it is noteworthy that *S. salivarius*, in our case, was also multi‐resistant and susceptible to only vancomycin, which proves that not all streptococci are susceptible to the first‐line antibiotics for neonatal sepsis. Prospective study of Corredoira et al. showed that *S. salivarius* had the greatest rate of resistance amongst the VGS to penicillin with 32%, in contrast to *S. bovis*, which were all susceptible to penicillin.^10^ In our case, upon receiving the primary report of Gram‐positive cocci, we initially kept our broad‐spectrum conventional antibiotics aiming for GBS; however, the outcome was not profitable. Therefore, vancomycin was used instead for 10 days, which showed a good result.

There are some limitations to our case report. It was more likely due to a vertical transmission since the bacteria were isolated in an early hemoculture in a symptomatic infant. However, since the birth was imminent, GBS screening, urine examination, and hemoculture were not done. In hindsight, it would have been better if our choice of antibiotics for the neonate had been oriented earlier by the isolation of maternal bacteria.

## 4 CONCLUSION

Though rare, *Streptococcus salivarius* should not always be considered as contaminant. Our case also strives to raise the awareness of the importance of routine hemoculture before prescribing antibiotics. It is always the gold standard for diagnosis in spite of a low rate of culture‐proven sepsis. Moreover, it is pivotal to track down antibiogram even in a familiar case scenario since Gram‐positive cocci are not always GBS until proven otherwise by hemoculture, and not all streptococci are susceptible to the first‐line antibiotics for neonatal sepsis.

## AUTHOR CONTRIBUTIONS

**Sakviseth Bin:** Conceptualization; investigation; visualization; writing – original draft; writing – review and editing. **Sethikar Im:** Supervision; writing – review and editing.

## FUNDING INFORMATION

There was no funding support for the case report.

## CONFLICT OF INTEREST

None declared.

## ETHICS STATEMENT

No ethical approval is required.

## CONSENT

Written consent was obtained from the patient's parents for the publication of this report.

## Data Availability

The data that support the findings of this study are available from the corresponding author upon reasonable request.
